# Dual-Level Probability Consistency FS-PFCE for Robust Transfer Classification of Rice Seed Vigor Using Near-Infrared Hyperspectral Imaging

**DOI:** 10.3390/plants15182818

**Published:** 2026-09-14

**Authors:** Xiaoping Wu, Yushuo Wang, Jianhao He, Yang Wang, Huan Liang, Liangquan Jia, Shenghui Wang

**Affiliations:** 1 School of Artificial Intelligence, Huzhou Normal University, Huzhou 313000, China; wuxipu@gmail.com (X.W.); 2024388322@stu.huznu.edu.cn (Y.W.); 2023388402@stu.huznu.edu.cn (J.H.); 2 College of Modern Agriculture, Zhejiang A&F University, Hangzhou 311300, China; wangyang@zafu.edu.cn; 3 Wuhan Academy of Agricultural Science, Wuhan 430065, China; lianghuanconf@126.com; 4 Jiyang College of Zhejiang A&F University, Zhuji 311800, China

**Keywords:** rice seed vigor, near-infrared hyperspectral imaging, transfer classification, Dual-Level Probability Consistency, cross-variety transfer

## Abstract

Rice seed vigor is an important indicator for evaluating seed quality and germination capability. Near-infrared hyperspectral imaging technology has shown great potential for seed vigor assessment. However, significant spectral distribution differences often exist among different rice varieties, resulting in degraded transfer classification performance. Moreover, under small-sample conditions, models have difficulty in sufficiently learning shared discriminative information among different varieties, leading to limited transfer generalization capability and restricting their application in cross-variety agricultural scenarios. In this paper, we propose a novel Dual-Level Probability Consistency FS-PFCE (DPC-FS-PFCE) model to achieve robust transfer classification of rice seed vigor across multiple varieties using near-infrared hyperspectral imaging. A total of 900 seed samples from three rice varieties were used, with 600 Chunyou83 samples serving as the master dataset and 150 samples each from Zhongzu53 and Zhongzao39 serving as slave datasets; transfer performance was evaluated on Zhongzu53, Zhongzao39, and their mixed dataset (MIX). Different probability consistency loss strategies were investigated to achieve joint utilization of global and local probability information. Furthermore, a multi-class weight direction consistency constraint was introduced to enhance parameter alignment and knowledge transfer between the master and slave models. The results demonstrated that the master model established using the Chunyou83 dataset achieved classification accuracies of 93.33%, 88.89%, and 87.78% on the Zhongzu53, Zhongzao39, and mixed MIX datasets, respectively, through the DPC-FS-PFCE transfer model. Compared with the Direct Transfer method, the proposed approach improved classification accuracy by 11.11%, 13.33%, and 8.89%, respectively. In addition, the proposed model exhibited more stable transfer classification performance under different master dataset proportions and complex data scenarios. The proposed DPC-FS-PFCE method effectively improves the cross-variety transfer classification capability for rice seed vigor assessment and provides a promising technical approach for nondestructive quality evaluation of crop seeds.

## 1. Introduction

Rice is one of the most important food crops worldwide and a major staple crop in China, contributing significantly to global food supply and the advancement of sustainable agriculture [1,2]. Seed vigor is an important indicator of seed quality, directly affecting seed germination rate, seedling growth, and final yield [3,4]. Therefore, achieving nondestructive and accurate detection of rice seed vigor is of great significance for elite seed selection and seed grading. Traditional seed vigor assessment methods mainly rely on standard germination tests, staining methods, and electrical conductivity measurements [5,6,7]. Although these methods provide relatively high accuracy, they suffer from several limitations, including long detection periods, high labor costs, and destructive procedures, making them unsuitable for large-scale rapid evaluation [8]. Near-infrared spectroscopy (NIRS) and hyperspectral imaging (HSI) technologies have been widely applied in agricultural product quality evaluation due to their advantages of rapidity, nondestructiveness, and the ability to acquire abundant spectral information [9,10,11,12,13]. Recent advances in machine learning and deep learning methods have further enhanced the capability of NIRS and HSI technologies to extract discriminative information from spectral data, enabling rapid nondestructive evaluation of crop seed quality [14,15,16]. However, in practical applications, significant spectral differences exist among rice seeds of different varieties, while variations in acquisition environments, instrument conditions, and batch effects can further induce considerable changes in data distributions. These cross-variety spectral differences are closely associated with variations in seed morphology, internal tissue structure, and biochemical composition. Differences in seed coat characteristics and internal structure may influence the scattering and propagation behavior of near-infrared radiation, whereas variations in moisture, starch, protein, and lipid contents can alter absorption features associated with O-H, C-H, and N-H bonds [14]. Previous studies have shown that characteristic spectral regions related to these molecular vibrations are distributed mainly within the near-infrared range, providing a physicochemical basis for cultivar-dependent spectral shifts [17,18]. Consequently, models developed for one rice variety may exhibit reduced generalization performance when directly applied to other varieties. Such cross-dataset distribution discrepancies result in significant performance degradation of models trained on a single dataset when applied to new datasets, becoming one of the key challenges limiting the practical application of NIR-HSI-based seed vigor detection technology. Therefore, improving the transfer generalization ability of models under varying data distribution conditions has become an important research direction in agricultural intelligent detection.

To address this issue, existing studies have mainly focused on deep transfer learning, domain adaptation, and domain generalization. Transfer learning provides a general framework for transferring knowledge across related datasets, tasks, or domains, while domain adaptation is a branch of transfer learning that aims to improve model performance on a different domain by leveraging knowledge from an available domain [19]. In contrast, domain generalization aims to learn models from available training domains that can generalize directly to previously unseen domains without accessing data from those domains during training [20]. Deep learning-based methods have improved cross-domain generalization through strategies such as feature transfer, parameter fine-tuning, feature distribution alignment, adversarial learning, and data augmentation. Wu et al. [21] combined NIR-HSI with a weighted loss deep convolutional neural network (DCNN) and assigned different weights to different categories for nondestructive rice seed vigor detection. Yang et al. [17] achieved a classification accuracy of 99.50% using a convolutional neural network, while the parameter-freezing-based transfer learning model achieved a cross-variety recognition accuracy of 98.75%, enabling rice seed vigor identification across different varieties. Rehman and Jin [22] introduced deep adversarial domain adaptation for hyperspectral calibration model transfer among plant phenotyping systems, using adversarial learning to reduce domain discrepancies between different hyperspectral imaging systems. Ye et al. [23] proposed a cross-domain mapping strategy based on Multi-CycleGAN for hyperspectral image classification, further demonstrating the potential of generative domain mapping for reducing distribution differences across domains. Furthermore, Zhou et al. [24] proposed the MixStyle method, which expands data distributions by mixing feature statistics of samples and alleviates cross-domain performance degradation to some extent. Qi et al. [25] established multiple convolutional neural network (CNN) models and introduced two transfer learning strategies, fine-tuning and MixStyle, to validate the effectiveness of MixStyle in knowledge transfer. Qi et al. [26] proposed the SAM-GAN model based on the classical Deep Convolutional Generative Adversarial Network (DCGAN) and introduced the spectral angle mapper (SAM) criterion into the loss function. By incorporating spectral waveform similarity constraints, the consistency between generated and real spectra was improved, thereby enhancing the capability of the model to generate high-quality spectral samples. Collectively, these studies demonstrate that deep transfer learning, domain adaptation, and domain generalization can alleviate cross-domain distribution discrepancies in agricultural sensing through representation transfer, feature alignment, and feature distribution expansion. Despite these advances, most existing approaches mainly focus on feature-level representation transfer, distribution alignment, or data augmentation, while the preservation of class-discriminative information during cross-domain transfer remains insufficiently explored. In addition, these methods often involve relatively complex network architectures and iterative optimization procedures, and their performance may be sensitive to the amount and distribution of available slave data. Therefore, achieving stable knowledge transfer under small-sample spectral classification conditions remains challenging.

Beyond deep transfer learning, domain adaptation, and domain generalization, spectral calibration transfer provides another perspective for addressing spectral distribution differences. Zhang et al. [27] proposed the Parameter-Free Framework for Calibration Enhancement (PFCE), which established correlation constraints between the master model and the slave model to achieve cross-domain spectral consistency modeling. Without manual parameter tuning, this framework effectively reduced data distribution differences and achieved promising performance in multiple spectral regression tasks. Subsequently, the PFCE framework was further extended by optimizing constraint conditions and introducing multi-task learning strategies [28], and its effectiveness was validated under cross-instrument and small-sample conditions [29]. Nevertheless, existing calibration transfer methods are mainly designed for continuous-variable regression tasks and lack explicit mechanisms for modeling multiclass probability distributions and preserving class-discriminative structures during transfer. Therefore, extending calibration transfer from regression-oriented modeling to multiclass classification while simultaneously maintaining knowledge consistency and class-discriminative information remains an important challenge for cross-variety spectral analysis. Accordingly, DPC-FS-PFCE is methodologically rooted in spectral calibration transfer rather than conventional deep domain adaptation. It extends the FS-PFCE framework from regression-oriented calibration transfer to multiclass spectral transfer classification by introducing dual-level probability consistency and class-discriminative parameter constraints between the master and slave models.

To address this problem, we propose the Dual-Level Probability Consistency Full-Supervised PFCE (DPC-FS-PFCE) model for multi-class transfer classification of rice seed vigor using NIR-HSI. The main contributions of this paper are summarized as follows:

1. A DPC-FS-PFCE model was developed for multi-class spectral transfer classification across multiple varieties, achieving robust transfer classification of rice seed vigor using NIR-HSI and providing a new approach for improving the cross-variety generalization ability of spectral transfer models.
2. A Dual-Level Probability Consistency (DPC) structure was constructed in the objective function, integrating Mean Probability Consistency (MPC) and Sample Probability Consistency (SPC), to provide a new solution for mitigating transfer performance degradation caused by spectral distribution differences among rice varieties.
3. Transfer strategies under different master dataset proportions were designed to achieve robust model transfer under limited-scale master dataset conditions, providing theoretical support for spectral transfer applications with small-sample data.

The organization of this paper is as follows. Section 2 describes the experimental sample preparation and data preprocessing methods in detail. Section 3 presents the theoretical foundation and algorithm derivation of the proposed model. Section 4 reports the model performance and experimental results. Section 5 further interprets the obtained results and discusses their implications. Section 6 summarizes the conclusions of this study.

## 2. Results

### 2.1. Performance Comparison

To evaluate the applicability of traditional classifiers in spectral transfer classification tasks, three representative machine learning classifiers, namely Random Forest (RF), Extreme Gradient Boosting (XGBoost), and Support Vector Classifier (SVC), adopted as baseline models. The classifiers were trained using the master dataset Chunyou83 and directly transferred to three different slave datasets for evaluation. All models were evaluated using the same test sets as those used for the DPC-FS-PFCE model, and the experimental results are presented in Table 1. The three slave datasets consisted of Zhongzu53, Zhongzao39, and MIX, which was constructed by combining the latter two datasets. For the Zhongzu53 slave dataset, RF, XGBoost, and SVC achieved accuracies of 84.44%, 84.44%, and 86.67%, respectively, among which SVC exhibited the best performance with an F1-score of 80.00%. However, when transferred to the Zhongzao39 and MIX slave datasets, all classifiers showed different degrees of performance degradation. Specifically, the accuracies of SVC on the Zhongzao39 and MIX datasets decreased to 75.56% and 80.00%, respectively. These results indicate that traditional classification models are highly affected by spectral distribution discrepancies between the master and slave datasets, making it difficult to maintain stable transfer classification performance. Therefore, a transfer calibration model specifically designed to alleviate spectral distribution differences is required.

### 2.2. Overall Transfer Classification Performance

To evaluate the effectiveness of the probability consistency loss in near-infrared hyperspectral transfer classification tasks, Master Model, Direct Transfer, Slave Model, and the proposed DPC-FS-PFCE method were compared. The experimental results are presented in Table 2. It can be observed that the DPC-FS-PFCE model achieved superior classification performance compared with Master Model, Direct Transfer, and Slave Model on all three slave datasets. These results indicate that the probability consistency constraint can effectively alleviate the performance degradation caused by spectral distribution discrepancies between the master and slave models, enhance the transferability of discriminative knowledge from the master model to the slave model, and improve cross-variety spectral transfer classification performance.

For the Zhongzu53 dataset, the Master Model achieved a classification accuracy of 79.44%, while Direct Transfer improved the accuracy to 82.22%, indicating that the discriminative knowledge learned by the master model possesses certain transferability. However, due to the existing spectral distribution differences between the master and slave datasets, directly transferring the model knowledge cannot fully exploit the discriminative capability of the master model. In contrast, the DPC-FS-PFCE model achieved an accuracy of 93.33%, which improved by 13.89%, 11.11%, and 8.89% compared with Master Model, Direct Transfer, and Slave Model, respectively. These results demonstrate that the DPC module effectively enhances the consistency of output probability distributions between the master and slave models, promotes the transfer of discriminative knowledge, and significantly improves the classification performance on the slave dataset. For the Zhongzao39 dataset, the DPC-FS-PFCE model also achieved the highest classification accuracy of 88.89%, representing an improvement of 13.33% compared with Direct Transfer 75.56%. This further demonstrates that when spectral differences exist among rice varieties, directly transferring the parameters of the master model is insufficient to achieve satisfactory classification performance, whereas introducing the probability consistency loss can effectively improve transfer classification performance. For the MIX dataset, which contains spectral information from both Zhongzu53 and Zhongzao39 varieties, the spectral distribution becomes more complex, increasing the difficulty of transfer classification. Despite this challenge, the DPC-FS-PFCE model still achieved an accuracy of 87.78%, demonstrating strong transfer stability and generalization capability.

Overall, the experimental results on the three slave datasets demonstrate that DPC-FS-PFCE achieved the best classification performance in cross-variety transfer classification tasks. These findings verify the effectiveness of the dual-level probability consistency constraint in improving spectral transfer classification performance. Furthermore, the proposed model maintained stable transfer performance under slave datasets with different levels of spectral distribution complexity, providing an experimental foundation for subsequent analysis of different probability consistency strategies.

**Figure 1:**
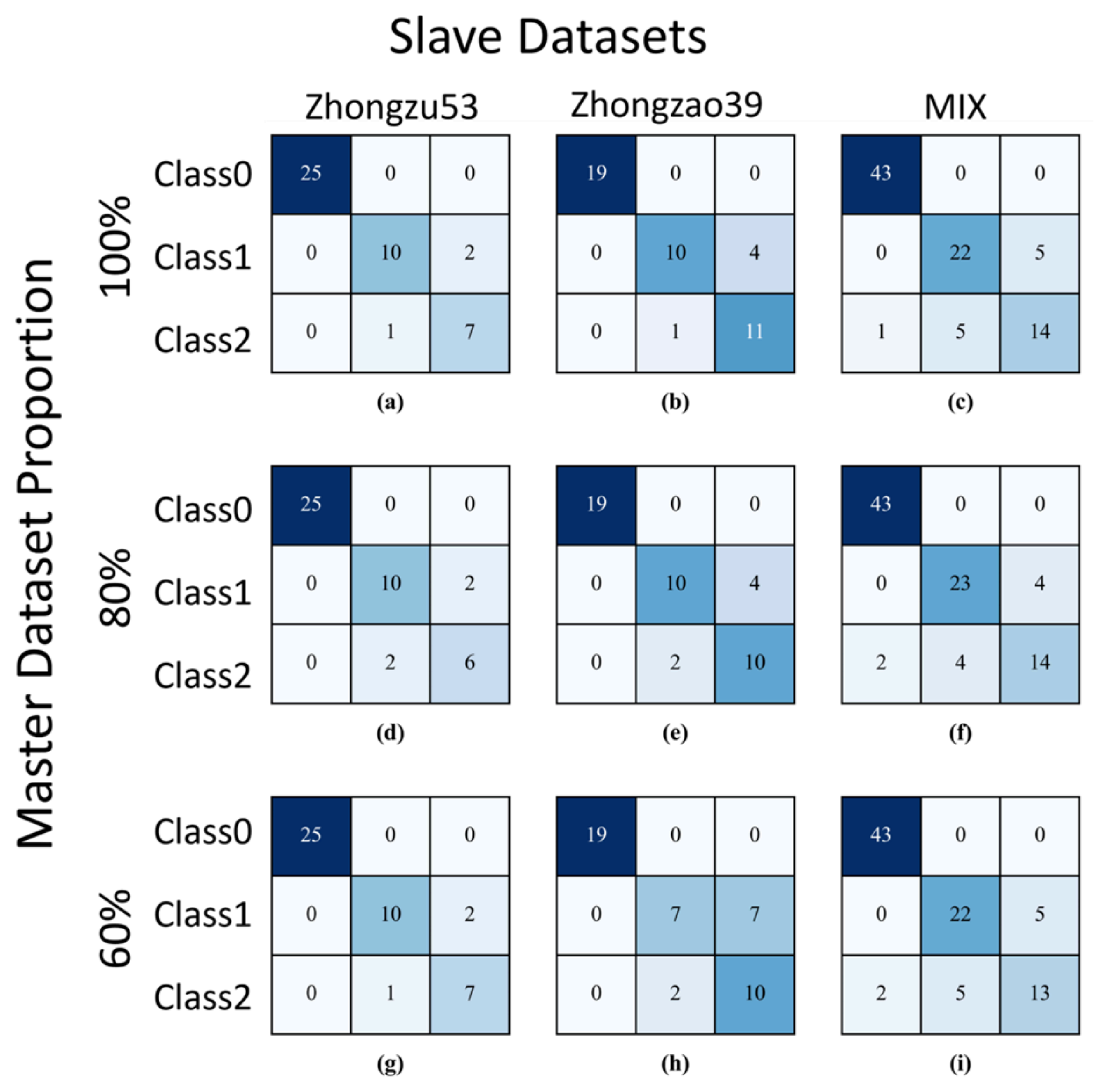
Confusion matrices of DPC-FS-PFCE under different transfer conditions: (**a**) Zhongzu53 with 100% master samples; (**b**) Zhongzao39 with 100% master samples; (**c**) MIX with 100% master samples; (**d**) Zhongzu53 with 80% master samples; (**e**) Zhongzao39 with 80% master samples; (**f**) MIX with 80% master samples; (**g**) Zhongzu53 with 60% master samples; (**h**) Zhongzao39 with 60% master samples; (**i**) MIX with 60% master samples. The color intensity represents the number of samples in each cell, with darker blue indicating a larger number of samples.

Figure 1 presents the confusion matrices of the DPC-FS-PFCE model under different master sample proportions and slave datasets. The model achieved reliable recognition performance for the three vigor categories under different transfer conditions, demonstrating its capability to preserve class-discriminative information during knowledge transfer. Class 0 (high-vigor seeds) exhibited consistently high recognition accuracy across all slave datasets and master sample proportions, with almost no misclassification observed. This indicates that high-vigor seeds possess relatively distinctive spectral characteristics, enabling effective discrimination from other vigor categories. Compared with Class 0, more confusion occurred between Class 1 (low-vigor seeds) and Class 2 (non-viable seeds), especially in the MIX dataset. This phenomenon can be attributed to the similar physiological states and spectral responses between low-vigor and non-viable seeds, resulting in partially overlapping spectral characteristics. For example, under the 100% master sample proportion condition, several Class 1 samples in the MIX dataset were misclassified as Class 2, while some Class 2 samples were incorrectly assigned to Class 1. When the master sample proportion decreased from 100% to 60%, the confusion between Class 1 and Class 2 slightly increased in some slave datasets, whereas Class 0 maintained stable recognition performance. These results indicate that DPC-FS-PFCE can preserve the major class-discriminative structures transferred from the master model, particularly under limited master sample conditions. By integrating global and sample-level probability consistency constraints, the proposed method improves the robustness of cross-variety seed vigor transfer classification.

### 2.3. Impact of Master Sample Proportion

To further evaluate the transfer performance of the model under small-sample conditions and compare the effectiveness of different probability consistency strategies in transfer classification tasks, 100%, 80%, and 60% of the master dataset samples were respectively selected to train the master models. The classification performance of MPC-FS-PFCE, SPC-FS-PFCE, and DPC-FS-PFCE on different slave datasets was compared, and the experimental results are presented in Table 3. By gradually reducing the proportion of master samples, the small-sample scenario with limited labeled samples in practical applications was simulated, allowing further evaluation of the adaptability of different probability consistency strategies for small-sample transfer tasks.

Overall, the three probability consistency strategies maintained relatively good transfer classification performance under different master sample proportions. However, as the master sample proportion decreased and the spectral distribution complexity of the slave datasets varied, performance differences among the strategies gradually emerged, indicating that probability consistency strategies play an important role in improving transfer performance under small-sample conditions. When the entire master dataset was used for model training 100%, the three strategies exhibited similar classification performance on the Zhongzu53 and Zhongzao39 datasets. In contrast, for the MIX dataset with a more complex class composition, the accuracies of SPC-FS-PFCE and DPC-FS-PFCE were both improved by 6.67% compared with MPC-FS-PFCE. This result indicates that, in complex transfer scenarios containing spectral information from multiple rice varieties, introducing sample-level probability consistency constraints can further enhance the model’s adaptability to spectral feature variations. When the master sample proportion was reduced to 80% and 60%, the discriminative information that could be learned by the master model was limited, increasing the difficulty of transfer classification. Under these conditions, MPC-FS-PFCE, SPC-FS-PFCE, and DPC-FS-PFCE were all able to maintain classification performance to some extent. However, DPC-FS-PFCE exhibited more stable transfer capability under different master sample proportions and slave dataset conditions.

The experimental results under different conditions indicate that different probability consistency strategies exhibit different applicability. However, as the number of master samples decreases, the difficulty of cross-variety spectral transfer classification gradually increases. Despite this challenge, DPC-FS-PFCE maintained stable and superior classification performance, demonstrating its effectiveness under small-sample conditions.

### 2.4. Robustness Analysis Under Different Master Sample Proportions

**Figure 2:**
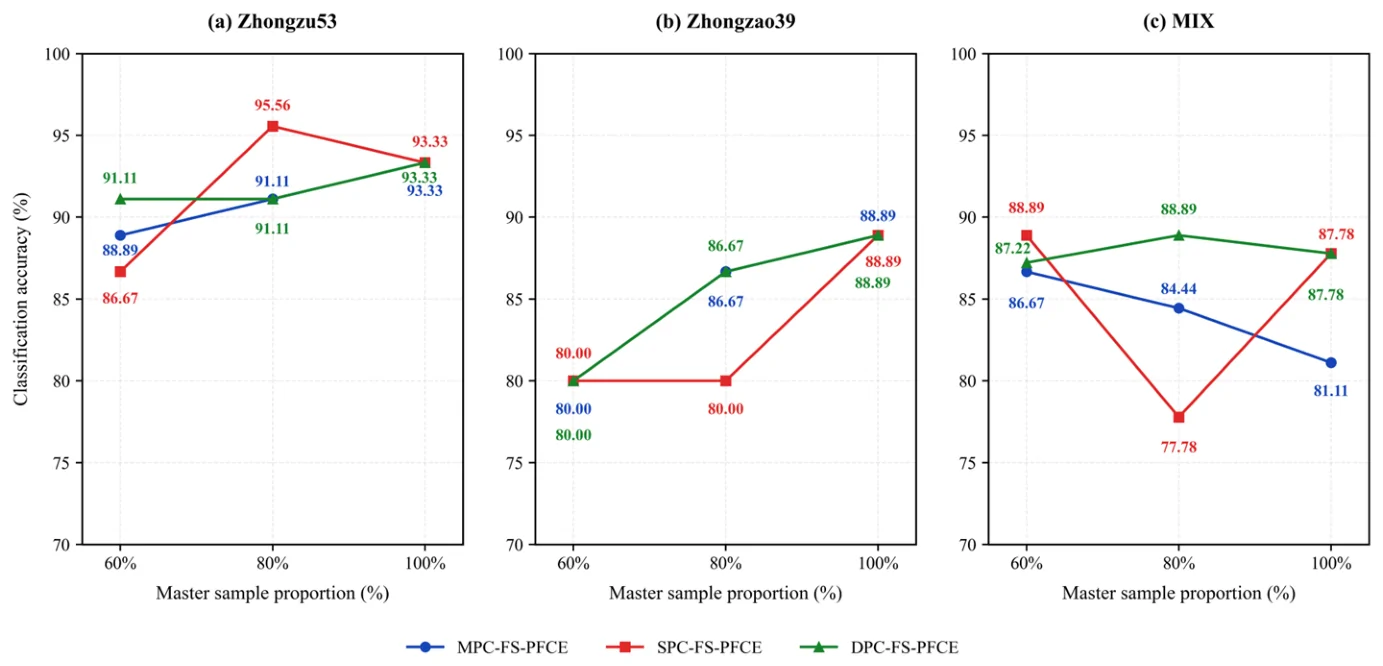
Transfer classification performance of three probability consistency strategies under different master sample proportions.

Figure 2 illustrates the changes in transfer classification performance of the three probability consistency strategies under different master sample proportions. Different slave spectral datasets exhibited varying sensitivities to changes in the master sample proportion. Nevertheless, DPC-FS-PFCE maintained stable performance across all three slave datasets, with consistently smaller fluctuations in classification accuracy.

For the Zhongzu53 dataset, as shown in Figure 2a, the classification accuracy of the three probability consistency strategies showed an overall increasing trend with the increase in the master sample proportion and gradually converged. This indicates that more master samples enabled the models to learn more sufficient discriminative information. This result also indicates that the amount of information available in the master dataset can influence the effectiveness of knowledge transfer. For Zhongzao39 Figure 2b, the three strategies exhibited relatively small variations, suggesting that the transfer task on this dataset was less affected by changes in the master sample size. In contrast, the MIX dataset Figure 2c, which contains spectral information from two rice varieties, exhibited more complex spectral variations, resulting in more evident performance differences among the three strategies.

Specifically, MPC-FS-PFCE showed a continuous decreasing trend as the master sample proportion changed, whereas SPC-FS-PFCE exhibited noticeable fluctuations. By comparison, DPC-FS-PFCE consistently maintained higher classification accuracy and showed the smallest variation range in performance.

The results from the three subfigures indicate that DPC-FS-PFCE maintained stable classification performance across different slave spectral datasets, master sample proportions, and transfer task complexities. By incorporating both MPC and SPC losses, the proposed model effectively integrates global and sample-level probability information, thereby improving its adaptability to small-sample transfer tasks and enhancing the stability and robustness of near-infrared hyperspectral transfer classification.

### 2.5. Interpretation Analysis of Transfer Spectral Features Learned by DPC-FS-PFCE

**Figure 3:**
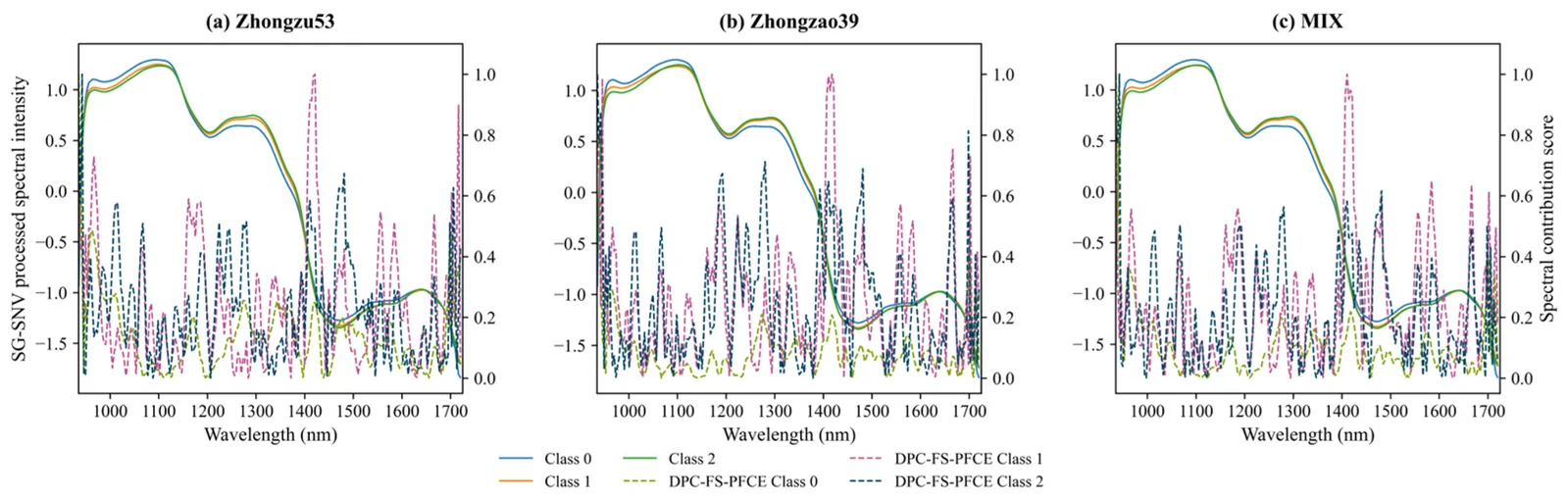
Class-specific spectral band contribution map of DPC-FS-PFCE.

To further investigate the discriminative spectral features learned by the DPC-FS-PFCE model during the transfer classification process, spectral contribution analysis was employed to interpret the model predictions on the Zhongzu53, Zhongzao39, and MIX datasets. As shown in Figure 3, different classes exhibited distinct spectral contribution distributions across different datasets, indicating that DPC-FS-PFCE can effectively extract key discriminative spectral regions according to class-specific characteristics.

For the Zhongzu53 dataset Figure 3a, the model mainly focused on the spectral regions of approximately 940–970 nm, 1180–1300 nm, 1400–1480 nm, and 1680–1700 nm. Among these regions, the relative contribution patterns differed among the three classes, with Class 1 showing a pronounced contribution around 1400–1430 nm, while Class 2 exhibited relatively high contributions at several wavelength regions across the measured spectral range. The Zhongzao39 Figure 3b and MIX Figure 3c datasets showed similar spectral contribution patterns. Similar high-contribution regions were observed in these datasets, although their relative contribution magnitudes varied among classes and datasets. Class 2 also exhibited relatively high contributions around 1248–1255 nm. Further analysis showed that relatively high spectral contributions were repeatedly observed around 940–970 nm, 1180–1300 nm, 1400–1480 nm, and 1680–1700 nm under different dataset conditions. Specifically, the region of approximately 940–970 nm is associated with higher-order O-H and C-H overtone absorptions and may reflect variations in moisture and organic constituents. The 1180–1300 nm region is commonly associated with C-H-related absorptions and may reflect variations in carbohydrates, lipids, and other organic storage compounds, whereas the 1400–1480 nm region is commonly associated with O-H and N-H overtone absorptions and may therefore be sensitive to variations in moisture, starch, and protein-related constituents. In addition, the 1680–1700 nm region is mainly related to C-H overtone absorption and may reflect changes in lipids and other organic storage substances.

These results indicate that DPC-FS-PFCE can preserve physiologically and biochemically meaningful spectral information associated with seed vigor across different rice varieties and mixed datasets, thereby providing a biochemical and spectral basis for the cross-variety transfer classification results.

### 2.6. Sensitivity Analysis of the Weighting Coefficient α

**Figure 4:**
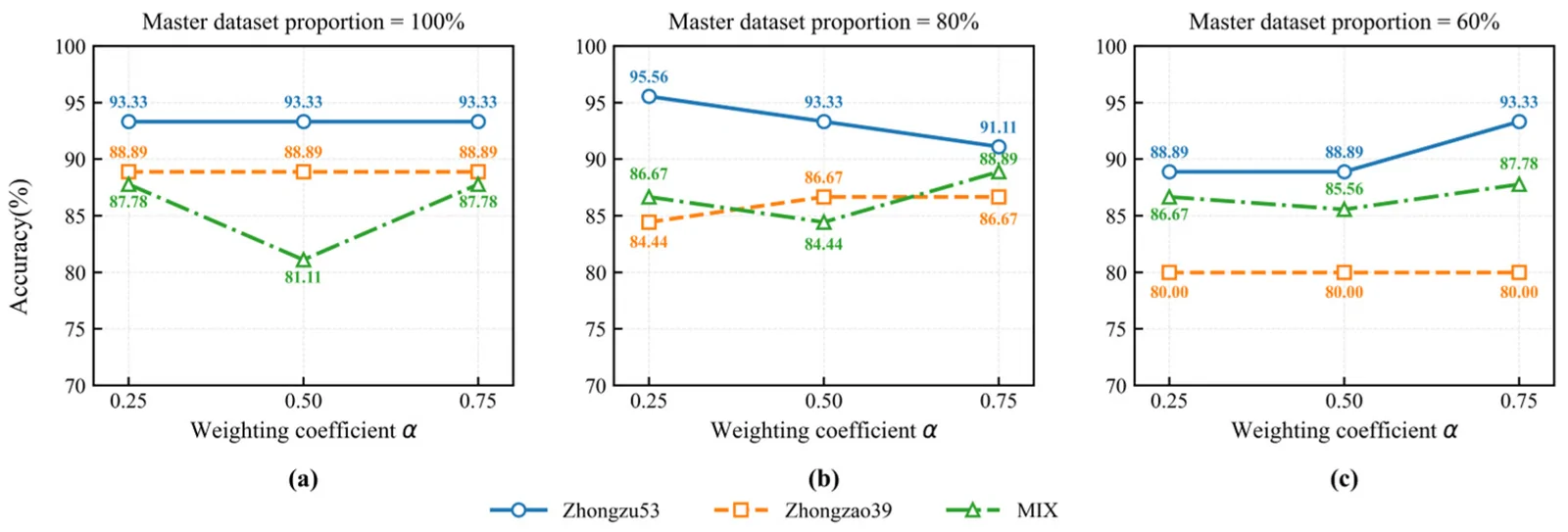
Classification accuracy of DPC-FS-PFCE under different weighting coefficient α and master dataset proportions: (**a**) 100% master dataset; (**b**) 80% master dataset; (**c**) 60% master dataset.

To investigate the influence of the weighting coefficient α in the DPC loss, sensitivity experiments were conducted by setting α to 0.25, 0.50, and 0.75 under different master dataset proportions. The transfer classification accuracies on Zhongzu53, Zhongzao39, and MIX are shown in Figure 4. Overall, the model exhibited different sensitivities to α across slave datasets and master sample proportions. For Zhongzu53, the classification accuracy remained relatively stable under most settings, while α = 0.75 achieved the highest accuracy of 93.33% when 60% of the master dataset was used. For Zhongzao39, the influence of α was generally limited, particularly under the 100% and 60% master dataset settings, whereas slight improvements were observed under the 80% setting. For MIX, the performance showed greater variation with α, reflecting the higher spectral heterogeneity introduced by combining two slave varieties.

These results are consistent with the design of the DPC strategy. In cross-variety transfer classification, the global probability distribution reflects the overall class-structure relationship between the master and slave models and therefore plays the primary role in maintaining transferable knowledge. In contrast, sample-level probability consistency mainly provides complementary local discriminative information. Cross-variety spectral shifts are mainly reflected in global distributions, while sample-level variations provide local complementary information. Accordingly, assigning a relatively larger weight to MPC while retaining the contribution of SPC enables the model to emphasize global distribution consistency while preserving useful sample-level details, thereby balancing the overall transfer structure with local discriminative information. The sensitivity analysis further supports this weighting scheme and the selection of α = 0.75.

## 3. Discussion

Rice seed vigor detection is essentially a fine-grained classification task involving physiological variations and spectral distribution changes. The main challenge does not lie in the existence of obvious overall spectral differences among different vigor classes, but rather in extracting discriminative features associated with vigor states from complex near-infrared spectral information and transferring such knowledge across different varieties. Due to differences in genetic background, tissue structure, and chemical composition among rice varieties, their spectral responses often exhibit significant distribution shifts, making classification models developed on a single variety difficult to directly apply to other varieties. Moreover, the acquisition of high-quality seed vigor samples is costly and time-consuming, further increasing the difficulty of transfer classification under small-sample conditions. Therefore, improving model generalization ability under cross-variety and small-sample scenarios is a critical issue for promoting the practical application of NIR-HSI technology in agriculture.

The FS-PFCE framework was originally developed for quantitative analysis of near-infrared spectroscopy, where spectral information transfer from the master model to the slave model is achieved through the objective function and regression coefficient correlation constraints [30]. However, seed vigor classification differs fundamentally from spectral quantitative prediction. Quantitative analysis mainly focuses on minimizing continuous variable prediction errors, whereas classification tasks rely more on establishing discriminative boundaries among categories and extracting class-related features. Consequently, optimization objectives designed for regression tasks cannot fully characterize the complex class-discriminative relationships involved in cross-variety rice seed vigor classification. To address this limitation, this study replaced the regression loss in FS-PFCE with a multi-class cross-entropy loss, transforming the transfer optimization process from continuous value fitting to class probability discrimination [31]. Meanwhile, the correlation constraint was retained to preserve effective structural information learned from the master model, providing a new framework for cross-variety seed vigor transfer classification.

Although parameter correlation constraints can facilitate structural information transfer between the master and slave models, parameter-level similarity alone cannot fully guarantee effective classification knowledge transfer. For classification models, the learned knowledge is not only embedded in model parameters or structures but also reflected in the class-related information and prediction confidence contained in output probability distributions. Knowledge distillation studies have demonstrated that soft probability outputs contain richer inter-class relationship information than hard labels and can serve as important carriers for knowledge transfer between models [32,33]. In addition, consistency regularization studies have shown that imposing consistency constraints on model predictions under different distributions can improve model stability against distribution variations [34].

Based on these findings, this study developed two probability consistency strategies, namely MPC and SPC. MPC maintains the global probability distribution consistency between the master and slave models, preserving the overall class structure relationship, whereas SPC constrains sample-level prediction probability variations to retain local discriminative information. For datasets with more heterogeneous spectral distributions, local probability information can provide additional discriminative cues, and the two strategies exhibit complementary effects under different transfer scenarios. Accordingly, the DPC strategy was proposed by integrating MPC and SPC to establish dual-level probability consistency constraints, thereby improving the adaptability of the model to complex spectral variations. The generally favorable performance of DPC may result from the simultaneous preservation of global class-distribution relationships and local sample-level discriminative information, thereby reducing the limitation of relying on a single level of probability alignment. The superior performance of DPC-FS-PFCE over RF, XGBoost, and SVC may be attributed to its explicit consideration of the spectral distribution discrepancy between the master and slave datasets. In contrast, conventional classifiers are trained only on the master dataset and do not incorporate dedicated transfer constraints, making them more sensitive to cross-variety spectral shifts. The experimental results further support this analysis, showing that DPC maintained stable transfer performance under different master sample proportions and slave spectral dataset complexities. This stability may be attributed to the dual-level probability consistency constraints, which preserve transferable class-distribution and sample-level discriminative information and thereby reduce the dependence of the transfer process on the size of the master dataset.

Although the DPC-FS-PFCE model achieved promising transfer classification performance, several limitations remain. First, model validation in this study was conducted on a limited number of rice varieties and seed vigor datasets. Future studies should further extend the evaluation to more varieties, different geographical origins, and more complex acquisition conditions to comprehensively assess the generalization capability of the proposed model [35]. Second, the vigor labels in this study were established primarily according to germination timing based on the standardized first and final germination-counting points specified in GB/T 3543.4-2025. While this approach provides a standardized and reproducible basis for distinguishing differences in germination speed and germination ability, seed vigor is inherently a multidimensional physiological trait. Future studies could integrate germination timing with additional individual-level physiological indicators, such as seedling length, root and shoot growth, biomass, or composite vigor indices, to establish a more comprehensive and biologically reliable ground-truth labeling system. Third, the current study employed fixed weights to combine MPC and SPC. However, the optimal balance between the two consistency constraints may vary under different data distributions. Future research could develop adaptive weight learning strategies based on data distribution characteristics, enabling dynamic adjustment of the probability consistency loss and further improving classification performance under complex spectral transfer scenarios. Fourth, although the full NIR-HSI spectrum was retained in this study to preserve comprehensive spectral information for cross-variety transfer modeling, the spectral contribution analysis indicated that discriminative information was mainly concentrated in several wavelength regions, including approximately 940–970 nm, 1180–1300 nm, 1400–1480 nm, and 1680–1700 nm. This suggests that the use of all 224 spectral bands may not be strictly necessary for practical rice seed vigor detection. However, because full-spectrum and reduced-band models were not systematically compared in the present study, it remains uncertain whether a small number of selected wavelengths can achieve comparable transfer performance. Future studies could therefore investigate wavelength-selection strategies and reduced-band modeling to evaluate whether classification accuracy and transfer robustness can be maintained while reducing spectral dimensionality and computational complexity. In this study, the mean spectrum of each seed ROI was used as the representative seed-level feature, providing a consistent model input while reducing the influence of pixel-level noise and intra-seed spatial variability. However, this strategy mainly exploits spectral information and does not fully utilize the spatial heterogeneity contained in hyperspectral images. In addition, the current spectral range did not cover 400–900 nm because an FX10 hyperspectral camera was unavailable. Future studies could further explore broader spectral ranges and spectral–spatial modeling to make fuller use of hyperspectral information and evaluate their potential for improving rice seed vigor classification and transfer performance. Finally, although spectral contribution analysis was performed to interpret the key wavelength regions utilized by the model and revealed the major spectral features involved in different transfer tasks, the current analysis mainly focused on feature importance. The evolution of spectral features during knowledge transfer from the master model to the slave model, as well as the mechanism by which probability consistency constraints influence discriminative information transfer, remain insufficiently explored. Future studies could combine advanced interpretable machine learning methods, feature attribution analysis, and visualization techniques for transfer processes to further investigate the decision-making mechanisms and cross-variety spectral knowledge transfer pathways of the proposed model.

## 4. Materials and Methods

**Figure 5:**
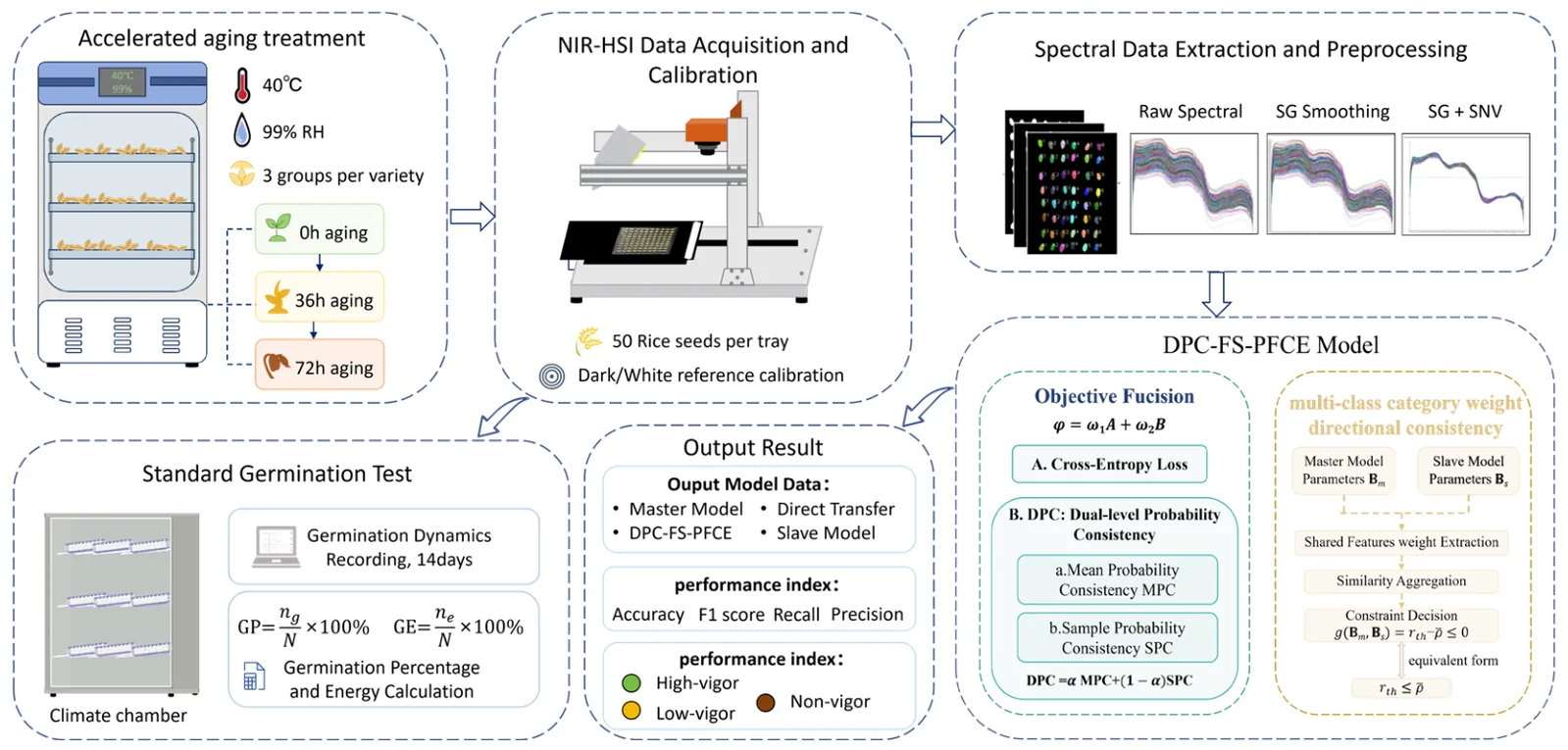
Experimental flow chart.

The overall experimental and analytical workflow of this study is illustrated in Figure 5, including accelerated aging treatment, NIR-HSI data acquisition and calibration, standard germination testing, spectral data extraction and preprocessing, and DPC-FS-PFCE modeling.

### 4.1. Sample Preparation

Three rice varieties, namely Chunyou83, Zhongzu53, and Zhongzao39, were selected as the research objects in this study from Huzhou City, Zhejiang Province, China. Since the storage conditions of seeds at different natural aging stages vary, the deterioration processes and vigor characteristics are difficult to maintain consistency. Moreover, naturally aged samples are difficult to obtain and have poor batch repeatability. Therefore, an artificial accelerated aging test was employed to simulate the natural aging process of seeds. Artificial accelerated aging is a standard seed vigor evaluation method recommended by the International Seed Testing Association (ISTA), which accelerates seed physiological deterioration under controlled high-temperature and high-humidity conditions, simulating changes during natural storage, including cell membrane damage, protein denaturation, and nucleic acid degradation, within a relatively short period. Although accelerated aging cannot completely reproduce the long-term natural aging process, both processes involve similar physiological deterioration mechanisms, such as oxidative stress, membrane damage, loss of cellular integrity, and changes in storage substances [36]. These physiological and biochemical changes can further alter near-infrared spectral responses associated with O-H, C-H, and N-H molecular vibrations through variations in moisture, proteins, lipids, and carbohydrates, thereby providing a physiological and spectral basis for using accelerated aging to generate seed samples with different deterioration levels [37,38]. Compared with natural aging, this method has advantages such as good repeatability and easy sample acquisition, and has therefore been widely applied in seed vigor research.

**Figure 6:**
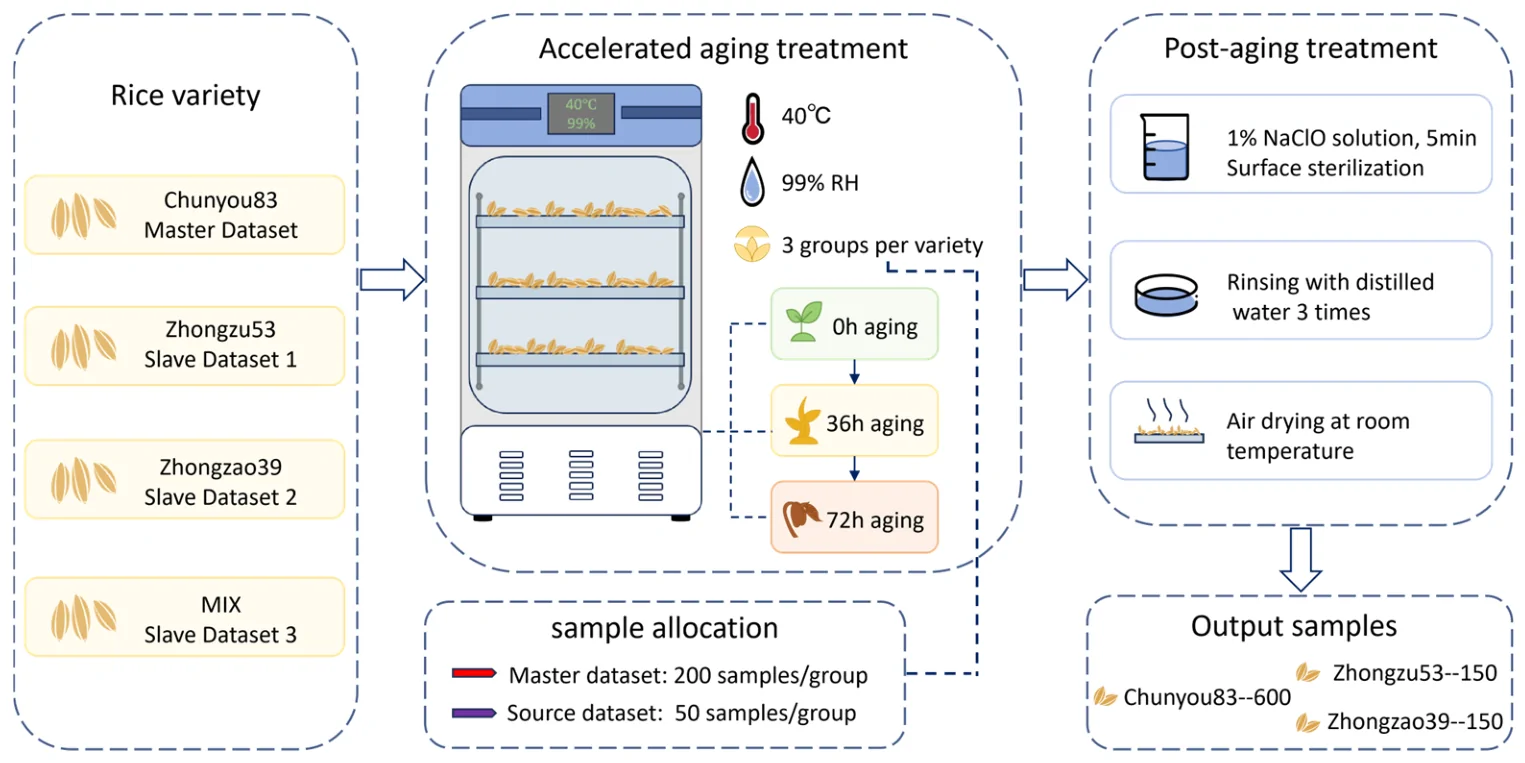
Sample preparation workflow.

The dataset collected from Chunyou83 was used as the master dataset, containing 600 samples, while datasets collected from Zhongzao39 and Zhongzu53 were used as slave datasets, with 150 samples collected for each variety. To obtain samples with different vigor levels, seeds of each variety were assigned to three artificial aging treatments with durations of 0, 36, and 72 h, respectively. Each aging treatment contained 200 samples for the master dataset and 50 samples for each slave dataset. The unequal sample sizes reflected the dataset configuration in this study, with the master dataset containing relatively more samples and the slave datasets containing fewer samples. This setting enabled the master model to learn relatively sufficient spectral and discriminative information while evaluating transfer performance under limited slave-sample conditions. The seeds were subjected to artificial accelerated aging in a climate chamber at 40 °C and 99% relative humidity. After aging treatment, the seeds were soaked in 1% sodium hypochlorite solution (Hangzhou Lionser Medical Disinfectant Co., Ltd., Jiande, China) for 5 min for surface disinfection, rinsed three times with purified water, and naturally dried at room temperature to obtain samples for subsequent experiments. The detailed sample preparation workflow is illustrated in Figure 6.

### 4.2. NIR-HSI Data Acquisition and Calibration

In this study, a near-infrared hyperspectral imaging system (Lab Scanner, Specim Spectral Imaging Ltd., Oulu, Finland) was employed to acquire hyperspectral data of rice seeds. The system mainly consisted of an FX17 near-infrared hyperspectral camera, halogen light sources, a moving platform, a computer, and corresponding control software. The FX17 camera covered a spectral range of 900–1700 nm with an average spectral resolution of 8 nm. The illumination system consisted of six 35 W halogen lamps symmetrically distributed on both sides of the camera. Before data acquisition, the hyperspectral imaging system was preheated for 30 min to ensure stable operation of the equipment.

During spectral data acquisition, rice seeds were randomly and evenly placed on a seed tray to ensure that individual seeds did not overlap or contact each other. The hyperspectral imaging system was controlled using the official LUMO-Scanner 2020 software. The vertical distance between the camera lens and the sample surface was approximately 25.2 cm. The moving platform speed was set to 24.70 mm/s, while the integration time and frame rate were maintained at 8.26 ms and 70 fps, respectively. Data acquisition was performed using a push-broom imaging mode, with 50 samples collected in each acquisition. Based on the platform speed and frame rate, the spatial sampling interval along the scanning direction was approximately 0.353 mm/line. To eliminate the effects of dark current and illumination non-uniformity, white and dark reference correction was performed. Before sample acquisition, a white reference image and a dark reference image were acquired using a white Teflon standard board and a lens cap, respectively. The corrected reflectance image was calculated according to Equation (1) as follows:

(1) $$R=\frac{I-B}{W-B}$$

where I represents the raw image, W represents the white reference image, B represents the dark reference image, and R represents the corrected reflectance image.

Following calibration, the whole seed was selected as the Region of Interest (ROI). The mean spectrum of all pixels within the ROI was extracted as the representative spectral feature of each seed. Finally, hyperspectral data of individual seeds were obtained, with each sample represented by a 1 × 224 dimensional spectral feature vector.

### 4.3. Standard Germination Test

The standard germination test was performed following the guidelines specified in GB/T 3543.4-2025 [39]. To establish a one-to-one correspondence between spectral data and germination results, each seed was assigned a unique identification number during spectral acquisition. Each group consisted of 50 seeds arranged in a 10 × 5 array. The row numbers were designated as A–J, and the column numbers were designated as 1–5. The seed located at the first row and first column was labeled as A1. The prepared experimental samples were placed into germination boxes according to their identification numbers. Each germination box was prelined with moistened germination paper at the bottom. The boxes were maintained in a climate chamber at a constant temperature of 30 °C for a 14-day standard germination test. Seed germination was recorded daily, and water was replenished when necessary. The first germination count was performed on the 5th day, and the final germination count was performed on the 14th day. The 5-day and 14-day thresholds used for vigor classification were based on the first and final germination-counting times specified in GB/T 3543.4-2025. Accordingly, germination timing relative to these two standardized assessment points was used to characterize differences in seed germination speed and germination ability [16]. Based on germination performance and germination time, seed samples were divided into three vigor levels. Seeds germinated within 5 days (≤5 days) were classified as high-vigor samples (Class 0), seeds germinated between 5 and 14 days (>5–14 days) were classified as low-vigor samples (Class 1), and seeds that remained ungerminated after 14 days were classified as non-vigorous samples (Class 2). Table 4 summarizes the germination numbers of rice seeds from different varieties at the three stages, together with their germination percentage (GP) and germination energy (GE). GP and GE were calculated according to Equations (2) and (3), respectively, as follows:

(2) $$\mathrm{GP}=\frac{n_{g}}{N}\times 100\%$$

(3) $$\mathrm{GE}=\frac{n_{e}}{N}\times 100\%$$

where $n_{g}$ represents the number of seeds germinated within 14 days, $n_{e}$ represents the number of seeds germinated within 5 days, and N represents the total number of seeds.

### 4.4. Spectral Data Preprocessing

**Figure 7:**
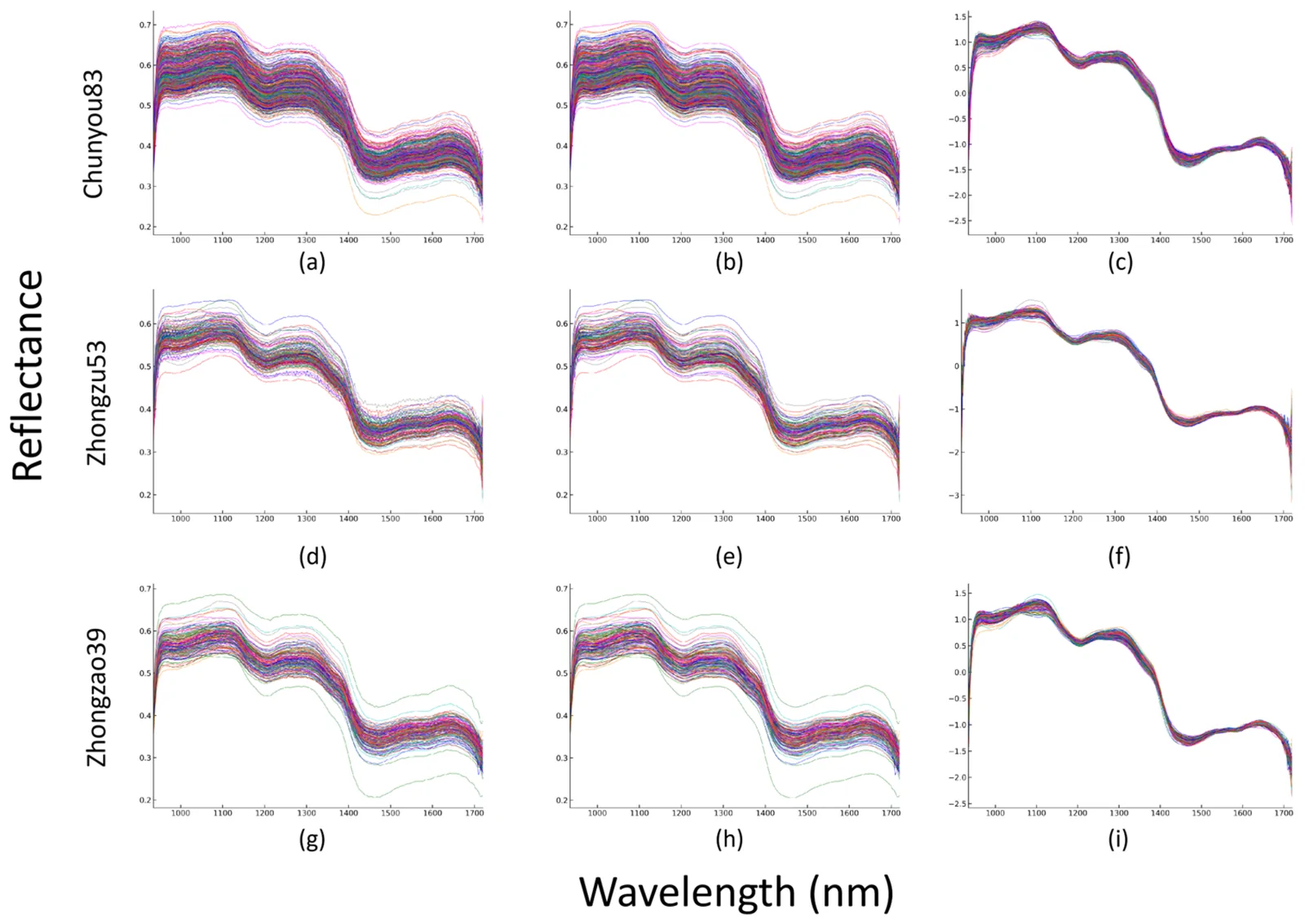
Raw and preprocessed spectral curves of different rice varieties: (**a**) raw spectra of Chunyou83; (**b**) SG-smoothed spectra of Chunyou83; (**c**) SG-SNV preprocessed spectra of Chunyou83; (**d**) raw spectra of Zhongzu53; (**e**) SG-smoothed spectra of Zhongzu53; (**f**) SG-SNV preprocessed spectra of Zhongzu53; (**g**) raw spectra of Zhongzao39; (**h**) SG-smoothed spectra of Zhongzao39; and (**i**) SG-SNV preprocessed spectra of Zhongzao39. Each colored line represents the spectral curve of an individual rice seed sample.

During hyperspectral data acquisition, raw spectra inevitably contain noise, baseline drift, and other interfering information due to variations in acquisition conditions, instrument noise, and illumination fluctuations. Therefore, spectral preprocessing is required before model development to reduce noise interference and preserve effective spectral features. Two preprocessing methods, Savitzky–Golay (SG) smoothing and Standard Normal Variate (SNV) transformation, were applied to the raw spectra. Figure 7 presents the raw spectral curves and the spectral curves obtained after different preprocessing procedures. Specifically, Figure 7a,d,g show the raw spectra of Chunyou83, Zhongzu53, and Zhongzao39, respectively. The spectra after SG smoothing are shown in Figure 7b,e,h, while the spectra after further SNV transformation are presented in Figure 7c,f,i.

#### 4.4.1. Savitzky–Golay Smoothing

SG smoothing is a spectral smoothing method based on the least-squares approach [40,41]. It performs smoothing by fitting a low-order polynomial within a moving window. The method can adapt to different signal characteristics and noise levels by adjusting the window size and polynomial order. It effectively suppresses random noise while preserving important spectral features, such as peak shape, peak position, and peak width. Therefore, SG smoothing has been widely applied in spectral data preprocessing. In this study, SG smoothing was performed using a window length of 15 and a polynomial order of 5.

#### 4.4.2. Standard Normal Variate Transformation

SNV transformation is a spectral normalization method that standardizes spectral data to a mean of zero and a standard deviation of one [42]. Without losing the original spectral information, SNV can enhance relevant spectral features, effectively eliminate non-specific measurement errors, and reduce the influence of interfering factors on spectral analysis. The SNV transformation is given in Equation (4) as follows:

(4) $$Z_{i}=\frac{X_{i}-\mu }{\sigma }$$

where $Z_{i}$ represents the spectral value at the i-th wavelength after SNV transformation, $X_{i}$ represents the original spectral value at the i-th wavelength, μ represents the mean value of all wavelength spectral values within the sample, and σ represents the standard deviation of the corresponding spectral values.

## 5. Theoretical Foundation and Algorithm Derivation of the Proposed Model

### 5.1. FS-PFCE

PFCE is a parameter-free framework for calibration enhancement that uses the correlation between the regression coefficients of the master model and slave model as a constraint without parameter tuning, thereby addressing deviations caused by variables such as instrument conditions and environmental variations. According to the availability of reference values, master spectra, and slave spectra, the PFCE framework can be applied in three scenarios: unsupervised PFCE (NS-PFCE), semi-supervised PFCE (SS-PFCE), and full-supervised PFCE (FS-PFCE). Among them, FS-PFCE is established when reference values, master spectra, and slave spectra are all available. It minimizes the prediction errors between the master model predictions and reference values, as well as those between the slave model predictions and reference values. Meanwhile, the correlation constraint of regression coefficients is introduced to transfer important features learned by the master model to the slave model, thereby constructing an enhanced slave model with improved prediction accuracy. The FS-PFCE framework mainly consists of two components: the objective function and the correlation constraint.

In the correlation constraint, $X_{m}$ and $X_{s}$ denote the master and slave spectral matrices, respectively; y represents the reference values of the standard samples. $b_{0,m}$ and $b_{0,s}$ are the intercept terms, and $b_{m}$ and $b_{s}$ are the regression coefficient vectors of the master and slave models, respectively. corr(·) represents the correlation coefficient. The correlation coefficient between $b_{m}$ and $b_{s}$ should be greater than the threshold $r_{th}$, ensuring the structural consistency of the regression coefficients between the two models. This constraint prevents excessive deviation of feature parameters between the slave model and the master model, thereby reducing the risk of overfitting.

The corresponding formulations of the objective function and correlation constraint are given as follows [27]:

$$\underset{b_{0,s},b_{s}}{\min}\left({\left\|y-[1\;X_{s}]\left[\begin{array}{c} b_{0,s}\\ b_{s} \end{array}\right]\right\|}^{2}+{\left\|[1{\;X}_{m}]\left[\begin{array}{c} b_{0,m}\\ b_{m} \end{array}\right]-[1{\;X}_{s}]\left[\begin{array}{c} b_{0,s}\\ b_{s} \end{array}\right]\right\|}^{2}\right)$$

(5) $$s.t.\;\mathrm{corr}(b_{s},b_{m})>r_{th}$$

As shown in Equation (5), the objective function of FS-PFCE consists of the sum of two loss terms. The term ${\left\|y-[1\;X_{s}]\left[\begin{array}{c} b_{0,s}\\ b_{s} \end{array}\right]\right\|}^{2}$ represents the residual error between the predictions of the slave model and the reference values y, which ensures the predictive performance of the slave model on the slave dataset. The term ${\left\|[1{\;X}_{m}]\left[\begin{array}{c} b_{0,m}\\ b_{m} \end{array}\right]-[1{\;X}_{s}]\left[\begin{array}{c} b_{0,s}\\ b_{s} \end{array}\right]\right\|}^{2}$ represents the residual squared error between the outputs of the master model and slave model. This term is used to maintain output consistency between the two models, thereby promoting knowledge transfer from the master model to the slave model.

### 5.2. Proposed DPC-FS-PFCE

The original FS-PFCE framework was mainly designed for spectral quantitative regression tasks. Its objective function adopts the mean squared error loss, and the model output consists of continuous prediction values, making it unsuitable for direct application to multi-class spectral transfer classification tasks. Moreover, its consistency constraint mainly considers the consistency of regression outputs and the correlation of regression coefficients, without effectively utilizing classification probability distribution information, thereby limiting the effective transfer of class-discriminative information. To overcome these limitations, the DPC-FS-PFCE model was developed for near-infrared hyperspectral transfer classification. The proposed model reconstructs FS-PFCE from quantitative regression to qualitative classification, providing a unified optimization framework for transfer classification of rice seed vigor across datasets from different rice varieties.

1. Objective function reconstruction: A Softmax output layer and multi-class cross-entropy loss were introduced to transform FS-PFCE from regression prediction to multi-class classification. Meanwhile, a Dual-Level Probability Consistency (DPC) loss combining Mean Probability Consistency (MPC) and Sample Probability Consistency (SPC) was developed to constrain the prediction probability distributions of the master and slave models, thereby enhancing classification knowledge transfer.
2. Correlation constraint reconstruction: A multi-class weight direction correlation constraint was introduced to replace the regression coefficient correlation constraint. By constraining the directional consistency of the corresponding class weight vectors between the master model and the slave model, the proposed method improves the transfer classification accuracy, stability, and robustness between rice seed vigor datasets from different varieties without requiring complex spectral calibration parameters.

The mathematical formulation of DPC-FS-PFCE consists of two components: the classification-based objective function and the multi-class weight direction consistency constraint. The detailed formulation and derivation are described as follows.

#### 5.2.1. Objective Function

For the multi-class classification problem, given a near-infrared hyperspectral dataset X(m×n), where m represents the number of samples and n represents the number of spectral variables, the corresponding labels are denoted as y(m×C), where C represents the number of classes. In this study, C=3. The objective of the classification task is to learn the mapping relationship from the spectral feature space to the class probability space. Therefore, for any sample $x_{i}(1\times n)$, linear mapping-based classification models are constructed separately in the master model and slave model, as defined in Equation (6):

(6) $$Z=\left[1X\right]\left[\begin{array}{c} b_{0}\\ B \end{array}\right]$$

where Z(m×C) represents the linear score matrix of the model, 1 denotes an m-dimensional column vector of ones, $b_{0}(1\times C)$ and B(n×C) represent the class-specific bias vector and feature weight matrix, respectively.

By introducing the Softmax function, the linear scores are transformed into a probability distribution. For the c-th class of the i-th sample, the predicted probability $p_{i,c}$ is defined in Equation (7) as follows:

(7) $$p_{i,c}=\frac{exp(z_{i,c}-\underset{j=1,\ldots ,C}{\max}z_{i,j})}{\sum_{j=1}^{C}exp(z_{i,j}-\underset{j=1,\ldots ,C}{\max}z_{i,j})}$$

where $z_{i,c}$ denotes the scalar element located at the i-th row and c-th column of matrix Z, representing the linear score of the c-th class for the i-th sample. j is the index of the class. The term $\underset{j=1,\ldots ,C}{\max}z_{i,j}$ represents the maximum linear score among all classes for the i-th sample. It is introduced to prevent numerical overflow in the exponential operation and ensure numerical stability during computation. The predicted probabilities of all samples form the probability matrix P(m×C).

In transfer classification modeling, NIR-HSI data of rice seeds from different varieties were collected. The dataset used for establishing the master model is denoted as the master spectral matrix $X_{m}$, while the dataset used for constructing the slave model is denoted as the slave spectral matrix $X_{s}$. After training the master model, the model parameters $b_{0,m}$ and $B_{m}$ were fixed, and only the slave model parameters $b_{0,s}$ and $B_{s}$ were optimized. Therefore, the objective function of the DPC-FS-PFCE model can be formulated in Equation (8) as follows:

(8) $$\underset{b_{0,s},B_{s}}{\min}\left\{\omega_{1}L_{CE}+{\omega_{2}L}_{DPC}\right\}$$

As can be observed, the objective function consists of two loss terms: the multi-class cross-entropy loss and the DPC loss. The parameters $\omega_{1}$ and $\omega_{2}$ denote the weighting coefficients of $L_{CE}$ and $L_{DPC}$ in the objective function, respectively.

(1) The multi-class cross-entropy loss $L_{CE}$ measures the classification error of the slave model on spectral samples, which is expressed in Equation (9) as follows:

(9) $$L_{CE}=-\frac{1}{m_{s}}\sum_{i=1}^{m_{s}}\sum_{c=1}^{C}y_{s,i,c}\log p_{s,i,c}$$

where $y_{s,i,c}$ denotes the one-hot encoded label of the i-th sample in the slave dataset. It enables only the corresponding true class to be selected during the loss calculation, ensuring that the slave model can effectively learn the mapping relationship between spectral features and class labels.

(2) The DPC loss is formulated as the weighted combination of the MPC loss and the SPC loss with a weighting factor α(α=0.75), which reduces the discrepancy between the probability distributions generated by the master and slave models. The MPC loss maintains the global probability distribution consistency, while the SPC loss maintains the local probability distribution consistency. The detailed calculation process is described in Equation (10) as follows:

(10) $$L_{DPC}={\alpha L}_{MPC}+\left(1-\alpha \right)L_{SPC}$$

(a) MPC Loss: The MPC loss is given in Equation (11):

(11) $$L_{MPC}=1-\frac{{{\bar{p}}_{m}}^{T}{\bar{p}}_{s}}{{\left\|\left.{\bar{p}}_{m}\right\|\right.}_{2}{\left.\left\|{\bar{p}}_{s}\right.\right\|}_{2}}$$

where ${\bar{p}}_{m}(1\times C)$ and ${\bar{p}}_{s}(1\times C)$ represent the average class probability vectors of all samples in the master and slave datasets, respectively. They are obtained by averaging the Softmax probability matrix P(m×C) along the sample dimensions, as defined in Equations (12) and (13):

(12) $${\bar{p}}_{m}=\frac{1}{m_{m}}\sum_{i=1}^{m_{m}}p_{m,i}$$

(13) $${\bar{p}}_{s}=\frac{1}{m_{s}}\sum_{i=1}^{m_{s}}p_{s,i}$$

(b) SPC Loss: The SPC loss is given in Equation (14):

(14) $$L_{SPC}=\frac{1}{m_{s}}\sum_{i=1}^{m_{s}}\left(1-\frac{{p_{m,i}}^{T}p_{s,i}}{{\left\|\left.p_{m,i}\right\|\right.}_{2}{\left.\left\|p_{s,i}\right.\right\|}_{2}}\right)$$

where $m_{m}$ and $m_{s}$ denote the numbers of samples in the master and slave datasets, respectively. Where $p_{m,i}(1\times C)$ and $p_{s,i}(1\times C)$ denote the class probability vectors generated by the master and slave models for the i-th prediction instance, respectively.

#### 5.2.2. Multi-Class Weight Direction Correlation Constraint

In the DPC-FS-PFCE model, the DPC loss ensures the consistency of output probability distributions between the master and slave models in the probability space. However, probability distribution consistency alone cannot guarantee the consistency of class-discriminative structures between the two models. Therefore, a multi-class weight direction correlation constraint is introduced into the parameter space to maintain consistent trends in spectral feature responses between the two models, thereby preserving similar classification decision directions. For the multi-class classification task in DPC-FS-PFCE, each class c corresponds to a class-specific weight vector $w_{c}$, where $w_{c}$ is the c-th column vector of the weight matrix B(n×C).

For the c-th class, let $w_{m,c}$ and $w_{s,c}$ denote the classification weight vectors of the master and slave models, respectively. Here $d=1,2,\;\ldots ,D_{share}$ represents the index of the feature dimension. $D_{share}$ denotes the dimension of the shared feature space, which is determined by $D_{share}=min\left(D_{m},D_{s}\right)$, where $D_{m}$ and $D_{s}$ denote the dimensions of the classification weight vectors in the master and slave models, respectively. $b_{m,c,d}$ and $b_{s,c,d}$ represent the classification weights corresponding to the d-th feature dimension for the c-th class in the master and slave models, respectively. Since the feature dimensions of the master and slave model datasets may be different, only the weights corresponding to the common feature space are aligned. The corresponding class weight vectors of the master and slave models are defined in Equations (15) and (16) as follows:

(15) $$w_{m,c}={\left[b_{m,c,d},b_{m,c,d},\ldots ,b_{m,c,d}\right]}^{T}$$

(16) $$w_{s,c}={\left[b_{s,c,d},b_{s,c,d},\ldots ,b_{s,c,d}\right]}^{T}$$

To ensure the consistency of decision boundaries across all classes, the cosine similarity of the weight vectors for each class is averaged over all classes to obtain the average class correlation, as defined in Equation (17):

(17) $$\bar{\rho }=\frac{1}{C}\sum_{c=1}^{C}\frac{{w_{m,c}}^{T}w_{s,c}}{{\left\|\left.w_{m,c}\right\|\right.}_{2}{\left.\left\|w_{s,c}\right.\right\|}_{2}}$$

Finally, the weight direction correlation constraint is formulated in Equation (18) as follows:

(18) $$g\left(B_{m},B_{s}\right)=r_{th}-\bar{\rho }\leq 0,$$

where $B_{m}$ and $B_{s}$ represent the classification weight matrices of the master and slave models, respectively.

**Figure 8:**
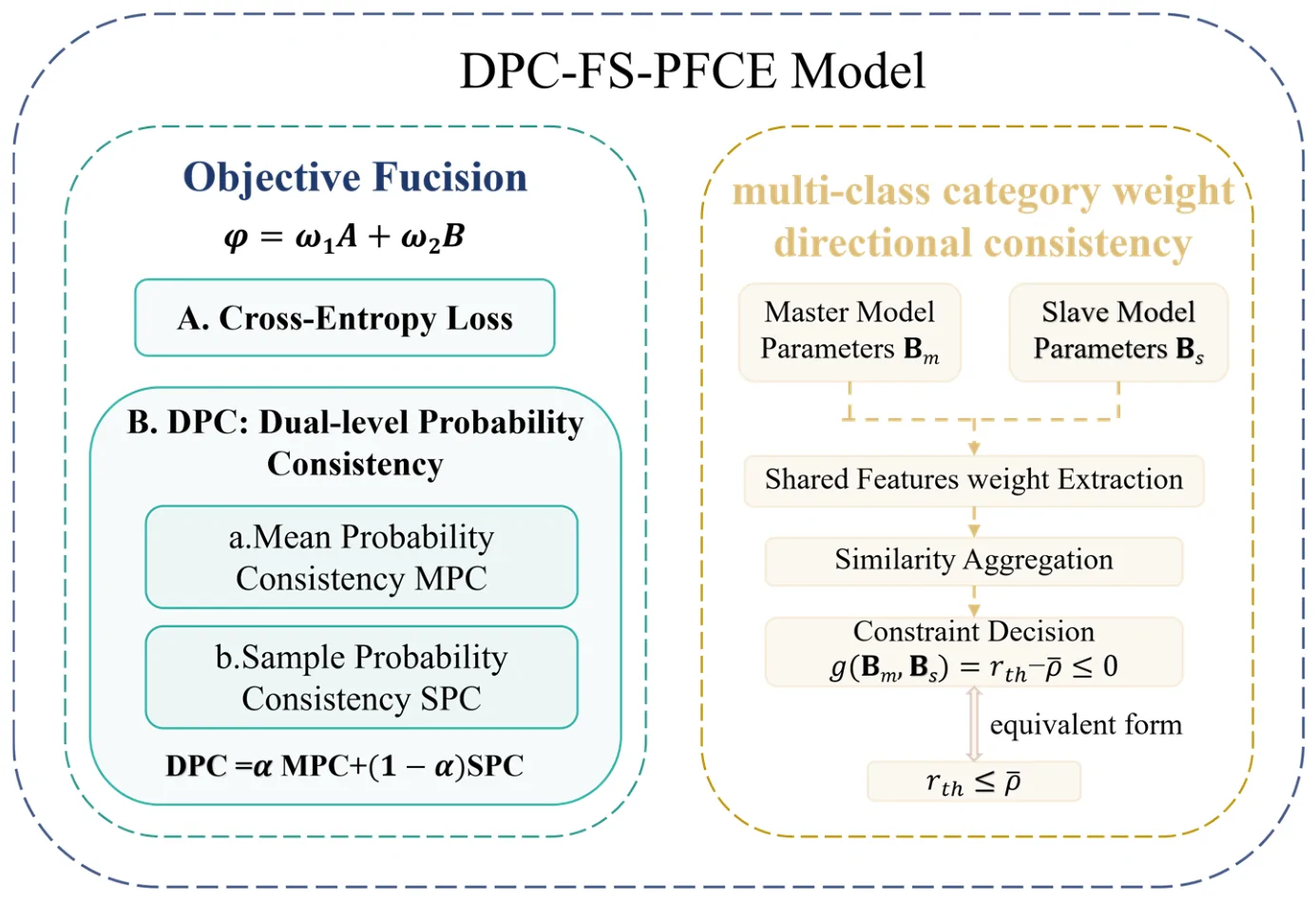
Architecture of the proposed DPC-FS-PFCE model.

The DPC-FS-PFCE model integrates the FS-PFCE framework with spectral transfer classification modeling for cross-variety rice seed vigor classification tasks. By introducing the Softmax function and multi-class cross-entropy loss, the learning objective is reconstructed from continuous regression optimization to class-discriminative optimization, thereby establishing a classification transfer framework based on probability distribution learning. The DPC module jointly models global class probability distributions and local prediction probability vectors, enabling consistency constraints between the master and slave models in the output space. Furthermore, the multi-class weight direction correlation constraint characterizes the consistency of class-discriminative structures in the parameter space, thereby maintaining stable decision boundaries during knowledge transfer. The detailed architecture of the proposed model is illustrated in Figure 8.

### 5.3. Implementation Details

The DPC-FS-PFCE model was optimized using the Sequential Least Squares Programming (SLSQP) algorithm implemented in SciPy. The maximum number of iterations was set to 500, with a function tolerance (ftol) of 1 × 10^−4^ and a finite-difference step size (eps) of 1 × 10^−4^. Since SLSQP performs constrained numerical optimization, no explicit learning rate was used. For Random Forest (RF), the number of trees was searched over {100, 200}, the maximum tree depth over {3, 5, 8}, the minimum number of samples required at a leaf node over {1, 3, 5}, and the number of features considered at each split over {sqrt, log2}. For XGBoost, n_estimators was searched over {50, 100}, learning_rate over {0.05, 0.1}, and max_depth over {2, 3}, while min_child_weight, reg_alpha, and reg_lambda were fixed at 5, 0.5, and 5, respectively. For the Support Vector Classifier (SVC), an RBF kernel was adopted, the penalty parameter ‘C’ was searched over {0.01, 0.1, 1, 10}, and ‘gamma’ was searched over {scale, 0.0001, 0.001, 0.01}. For all three classifiers, five-fold stratified cross-validation with data shuffling and a random seed of 42 was used during grid search, and the parameter combination yielding the highest macro-averaged F1-score was selected as the final model.

All experiments were implemented in Python 3.8.20 (Python Software Foundation, Beaverton, OR, USA) on a computer running Windows 11 Pro (Microsoft Corporation, Redmond, WA, USA), equipped with a 12th Gen Intel Core i5-12600KF CPU and 32 GB RAM. The computer was also equipped with an NVIDIA GeForce RTX 3060 GPU (NVIDIA Corporation, Santa Clara, CA, USA) with 12 GB dedicated memory. The main software packages included NumPy 1.24.3, pandas 2.0.3, SciPy 1.10.1, scikit-learn 1.3.0, and XGBoost 2.1.4.

### 5.4. Evaluation Metrics

The classification performance was evaluated using Accuracy, Precision, Recall, and F1-score. Accuracy represents the overall proportion of correctly classified samples in the test set and was calculated according to Equation (19) as follows:

(19) $$Accuracy=\frac{N_{correct}}{N_{total}}$$

where $N_{correct}$ denotes the number of correctly classified samples and $N_{total}$ denotes the total number of samples in the test set.

For the three-class seed vigor classification task, Precision, Recall, and F1-score were calculated using macro-averaging across all classes, as defined in Equations (20)–(22):

(20) $${Precisiom}_{macro}=\frac{1}{C}\sum_{c=1}^{C}\frac{TP_{c}}{TP_{c}+FP_{c}}$$

(21) $${Recall}_{macro}=\frac{1}{C}\sum_{c=1}^{C}\frac{TP_{c}}{TP_{c}+FN_{c}}$$

(22) $${F1}_{macro}=\frac{1}{C}\sum_{c=1}^{C}\frac{2P_{c}R_{c}}{P_{c}+R_{c}}$$

where C=3 represents the number of seed vigor classes; $TP_{c}$, $FP_{c}$, and $FN_{c}$ denote the numbers of true-positive, false-positive, and false-negative samples for class c, respectively; and $P_{c}$ and $R_{c}$ denote the Precision and Recall of class c. Therefore, the Accuracy reported in this study represents the overall classification accuracy of the corresponding test set.

## 6. Conclusions

In this paper, the DPC-FS-PFCE model was successfully applied to the cross-variety transfer classification task of rice seed vigor. The proposed model effectively alleviated the influence of spectral distribution discrepancies among different rice varieties on transfer performance, thereby improving classification stability and generalization capability on slave datasets. Regarding model design, a Softmax output layer and multi-class cross-entropy loss were introduced to establish a unified optimization framework for spectral transfer classification. Meanwhile, the DPC strategy was developed by integrating MPC and SPC with fixed weights, enabling joint modeling of global class structure information and local sample-level discriminative information, thereby enhancing cross-domain knowledge transfer capability. In addition, the multi-class weight direction consistency constraint maintained consistent class-discriminative structures between the master and slave models in the shared feature space, further improving transfer stability from the parameter perspective.

Experimental results demonstrated that under conditions involving higher spectral complexity and limited master model samples, the DPC-FS-PFCE model consistently outperformed Master Model, Direct Transfer, and Slave Model in overall classification performance, while maintaining strong stability and robustness across different experimental settings. Although the effectiveness of DPC-FS-PFCE was validated for cross-variety rice seed vigor transfer classification, the current study was mainly conducted using a limited number of rice varieties under a single hyperspectral acquisition condition. Future studies should further evaluate the applicability of the proposed model using more varieties, larger-scale datasets, and more complex data distributions. Overall, DPC-FS-PFCE provides a new solution for cross-variety classification of near-infrared hyperspectral data by integrating parameter structure transfer with probability consistency constraints. The results demonstrate that stable transfer classification can be achieved under limited labeled samples by exploiting the shared features and distribution information between master and slave models. This study provides a potential technical pathway for small-sample agricultural hyperspectral seed vigor detection and transfer learning applications, and offers valuable insights for future research on probability consistency-based spectral transfer learning.

## Figures and Tables

**Table 1:** Performance comparison of traditional classifiers under direct transfer.

Master Dataset	Slave Dataset	Model	Acc (%)	F1-Score (%)	Precision (%)	Recall (%)
Chun you83	Zhongzu53	RF	84.44	75.11	75.46	75.00
		XGBoost	84.44	76.70	77.13	76.39
		SVC	86.67	80.00	81.94	81.94
	Zhongzao39	RF	73.33	70.38	71.12	70.3
		XGBoost	73.33	69.79	70.00	69.79
		SVC	75.56	72.53	72.71	72.45
	MIX	RF	74.44	65.79	66.3	65.99
		XGBoost	75.56	66.99	67.27	67.22
		SVC	80.00	75.52	75.56	75.70

**Table 2:** Transfer classification performance of different models on three slave datasets.

Dataset	Model	Acc (%)	F1-Score (%)	Precision (%)	Recall (%)
Chunyou83	Master Model	79.44	79.51	79.92	79.44
Zhongzu53	Direct Transfer	82.22	81.64	81.59	82.22
	Slave Model	84.44	84.56	85.99	84.44
	DPC-FS-PFCE	93.33	93.38	93.63	93.33
Zhongzao39	Direct Transfer	75.56	74.21	76.72	75.56
	Slave Model	77.78	76.11	82.81	77.78
	DPC-FS-PFCE	88.89	88.84	90.06	88.89
MIX	Direct Transfer	78.89	75.85	78.48	78.89
	Slave Model	80.00	80.09	80.30	80.00
	DPC-FS-PFCE	87.78	87.73	87.72	87.78

**Table 3:** Transfer classification performance comparison under different master sample proportions.

Master Ratio	Slave Dataset	MPC-FS-PFCE (%)		SPC-FS-PFCE (%)		DPC-FS-PFCE (%)	
		Acc	F1-Score	Acc	F1-Score	Acc	F1-Score
100%	Zhongzu53	93.33	93.34	93.33	93.34	93.33	93.34
	Zhongzao39	88.89	88.84	88.89	88.84	88.89	88.84
	MIX	81.11	80.68	87.78	87.73	87.78	87.73
80%	Zhongzu53	91.11	91.11	95.56	95.56	91.11	91.11
	Zhongzao39	86.67	86.67	80.00	79.55	86.67	86.67
	MIX	84.44	84.15	77.78	77.90	88.89	88.62
60%	Zhongzu53	88.89	88.97	86.67	86.41	91.11	91.20
	Zhongzao39	80.00	79.55	80.00	79.55	80.00	79.55
	MIX	86.67	86.34	88.89	88.74	87.78	87.40

**Table 4:** Distribution of seed vigor classes and germination parameters.

Parameters	Chunyou83	Zhongzao39	Zhongzu53
Class 0	255	64	81
Class 1	175	39	36
Class 2	170	47	33
GP (%)	71.67	68.67	78
GE (%)	42.5	42.67	54

Note. GP, germination percentage; GE, germination energy. Class 0, high-vigor seeds (≤5 days); Class 1, low-vigor seeds (>5–14 days); Class 2, non-vigorous seeds (ungerminated after 14 days).

## Data Availability

The data presented in this study are available from the corresponding author upon request.

## Abbreviations

The following abbreviations are used in this manuscript:

NIRS	Near-infrared spectroscopy
HSI	hyperspectral imaging
ROI	Region of interest
GP	Germination percentage
GE	Germination energy
CNN	Convolutional neural network
DCNN	Deep convolutional neural network
SAM-GAN	Spectral angle mapper- Generative adversarial network
DCGAN	Deep convolutional generative adversarial network
NS-PFCE	Unsupervised-Parameter-free calibration enhancement
SS-PFCE	Semi-supervised-Parameter-free calibration enhancement
FS-PFCE	Full-supervised-Parameter-free calibration enhancement
DPC	Dual-level probability consistency
MPC	Mean probability consistency
SPC	Sample probability consistency
DPC-FS-PFCE	Dual-Level Probability Consistency FS-PFCE
